# In vitro induction of tetraploids and their phenotypic and transcriptome analysis in *Glehnia littoralis*

**DOI:** 10.1186/s12870-024-05154-w

**Published:** 2024-05-22

**Authors:** Xin Zhang, Ziyu Zheng, Jing Wang, Yuwen Li, Yan Gao, Lixia Li, Yujuan Pang, Fuhua Bian

**Affiliations:** 1 College of Life Science, Yantai University, Yantai, Shandong 264005 China; 2 Kunyushan Forest Farm, Yantai, Shandong 264112 China

**Keywords:** Tetraploid, *Glehnia littoralis*, Leaf, Phenotype, Transcriptome

## Abstract

**Background:**

*Glehnia littoralis* is a medicinal and edible plant species having commercial value and has several hundred years of cultivation history. Polyploid breeding is one of the most important and fastest ways to generate novel varieties. To obtain tetraploids of *G. littoralis* in vitro, colchicine treatment was given to the seeds and then were screened based on morphology, flow cytometry, and root tip pressing assays. Furthermore, transcriptome analysis was performed to identity the differentially expressed genes associated with phenotypic changes in tetraploid *G. littoralis*.

**Results:**

The results showed that 0.05% (w/v) colchicine treatment for 48 h was effective in inducing tetraploids in *G. littoralis*. The tetraploid *G. littoralis* (2n = 4x = 44) was superior in leaf area, leaf thickness, petiole diameter, SPAD value (Chl SPAD), stomatal size, epidermal tissues thickness, palisade tissues thickness, and spongy tissues thickness to the diploid ones, while the stomatal density of tetraploids was significantly lower. Transcriptome sequencing revealed, a total of 1336 differentially expressed genes (DEGs) between tetraploids and diploids. Chromosome doubling may lead to DNA content change and gene dosage effect, which directly affects changes in quantitative traits, with changes such as increased chlorophyll content, larger stomata and thicker tissue of leaves. Several up-regulated DEGs were found related to growth and development in tetraploid *G. littoralis* such as *CKI*, *PPDK*, *hisD* and *MDP1*. KEGG pathway enrichment analyses showed that most of DEGs were enriched in metabolic pathways.

**Conclusions:**

This is the first report of the successful induction of tetraploids in *G. littoralis*. The information presented in this study facilitate breeding programs and molecular breeding of *G. littoralis* varieties.

**Supplementary Information:**

The online version contains supplementary material available at 10.1186/s12870-024-05154-w.

## Background

*Glehnia littoralis*, belonging to the genus *Glehnia* in the family Apiaceae, is used in traditional medicine in Shandong Province, China, and has been cultivated for more than five hundred years. The leaves and roots of *G. littoralis* are edible while dried roots are used as medicine and has been listed in the Chinese and Japanese pharmacopoeias [1]. In addition, *G. littoralis* is a common halophytic plant that thrives in highly saline soils unsuitable for typical terrestrial flora [2]. In other words, *G. littoralis* combines medicinal, edible, economic and ecological values.

Due to habitat destruction and overexploitation, the wild resources of *G. littoralis* are endangered, and the market supply is mostly met from annual cultivated plants. During the process of long-term artificial cultivation, genetically consistent cultivars haven’t been developed to date. It is cultivated by seeds and the quality of *G. littoralis* is variable because it is a cross-pollinating plant. Therefore, it is imperative to develop varieties of *G. littoralis* with high yields to meet the high market demand and maintain species abundance.

Polyploidization is an important breeding strategy for improving valuable traits in plants [3, 4]. In comparison to conventional breeding methods, the artificial induction of polyploidy accelerates the breeding process and facilitates the development of novel and superior cultivars [5]. Colchicine, a widely utilized mutagen in plants [6], can be employed to increase the mutagenic efficiency by the application of high concentrations for short exposure durations or low concentrations for extended periods [7]. Presently, plant ploidy breeding has found application in the breeding of numerous medicinal plants, such as *Panax ginseng* [8], *Scutellaria baicalensis* [9] and *Plantago psylliumis* [7]. Polyploid plants typically exhibit distinct morphological traits and enhanced vigor in comparison to diploid plants. These traits include alterations in plant size, leaf size and thickness, modifications in developmental rate and cell size, and enhanced stress tolerance [10–12]. Phenotypic changes may be attributed to alterations in the expression of specific genes following chromosome doubling [13, 14], which has attracted attention from researchers. RNA sequencing technology can provide a large amount of genetic information that can reveal changes in the biochemical and regulatory aspects of plant secondary metabolism [15], reflecting the impact of gene dosage effects on phenotype and adaptability [16].

The lack of research on breeding efforts for *G. littoralis* has hindered the development of its industrialization. Artificial polyploidization is a relatively fast and effective way of plant breeding, but it has not been applied to *G. littoralis.* Moreover, *G. littoralis* is a monotypic genus species of Umbelliferae with a somatic cell chromosome number of 22 (2n = 2x = 22) [17]. The relatively low chromosome base facilitates artificial doubling of its chromosomes.

In this study, we induced chromosome doubling of *G. littoralis* in vitro with colchicine and identified tetraploid, then measured phenotypic characteristics and performed transcriptome sequencing of diploid and tetraploid *G. littoralis*. This study aimed to (1) obtain tetraploid *G. littoralis*, (2) investigate the phenotypic variation between diploids and tetraploids, and (3) to understand the changes in gene expression associated with phenotypic alterations. Overall, these findings will provide a theoretical basis for achieving speed breeding of *G. littoralis*.

## Results

### Induction of polyploidy *G. littoralis*

**Fig. 1:**
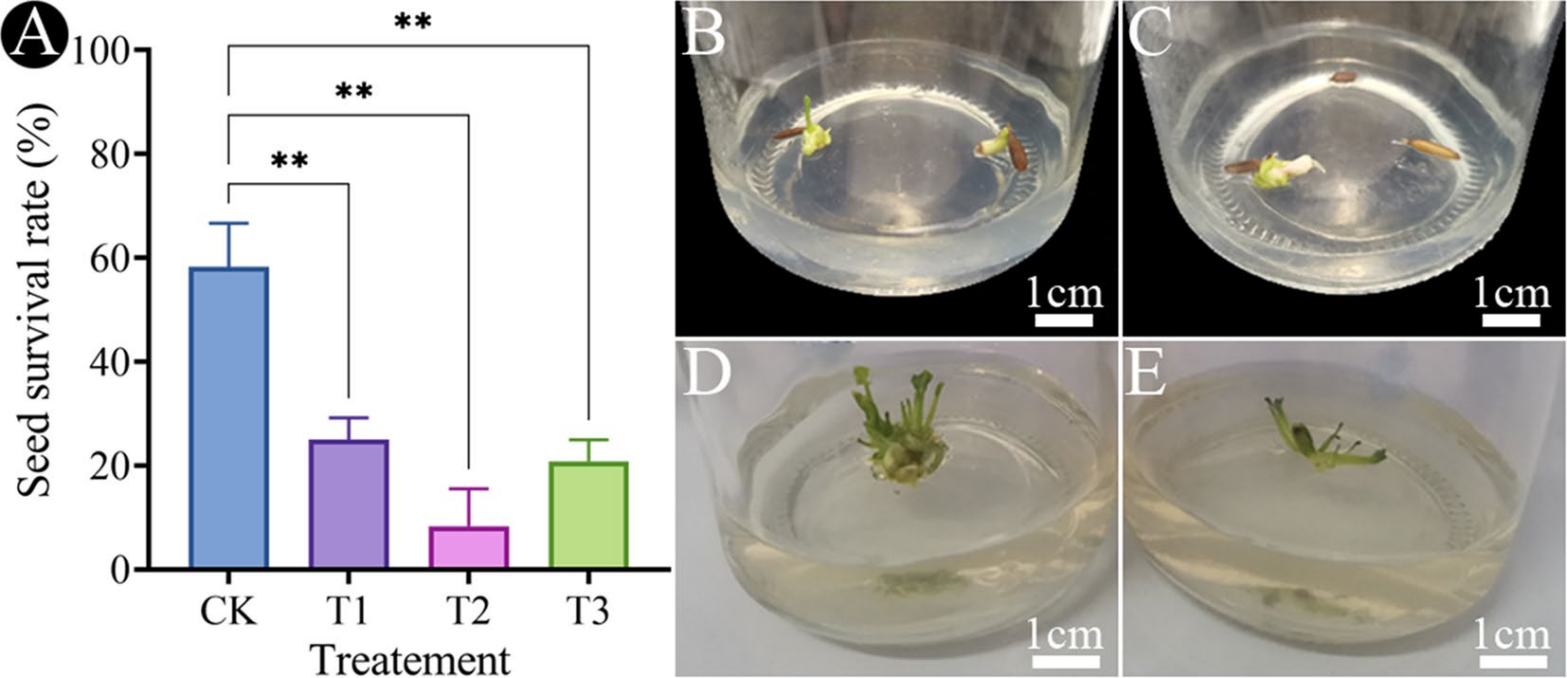
The induction of polyploidy *G. littoralis* from germinated seeds. (**A**) The survival rate of the seeds of *G. littoralis* on the proliferation medium. (**B**) Adventitious shoots regenerated from seeds after culture for 30 days (control). (**C**) Adventitious shoots regenerated from colchicine-treated seeds after culture for 30 days (T1). (**D**) Adventitious shoots regenerated from seeds after culture for 60 days (control). (**E**) Adventitious shoots regenerated from colchicine-treated seeds after culture for 60 days (T1). *p < 0.05, **p < 0.01. *Note* CK: control; T1: 0.05% (w/v) colchicine solution soaked seeds for 48 h; T2: 0.1% (w/v) colchicine solution soaked seeds for 24 h; T3: 0.15% (w/v) colchicine solution soaked seeds for 16 h

The survival rates of seeds treated with different treatments of colchicine were 25.0%, 8.3%, and 20.8%, respectively, which were significantly lower compared to the control on proliferation medium (Fig. 1A). And the most obvious swelling of adventitious shoots was observed when the seeds initially treated with 0.05% (w/v) colchicine for 48 h. After 30 days of culture, adventitious shoots began to appear. The adventitious shoots from the colchicine-treated seeds emerged approximately one week later than those from the control. (Fig. 1B and C). After culture for 60 days, many adventitious shoots became thicker and stronger around the colchicine-treated seeds than those from the controls. Apparently, the rate of proliferation of adventitious shoots from colchicine-treated seeds was slower than that from the control (Fig. 1D and E).

### Grading and screening of *G. littoralis*

**Fig. 2:**
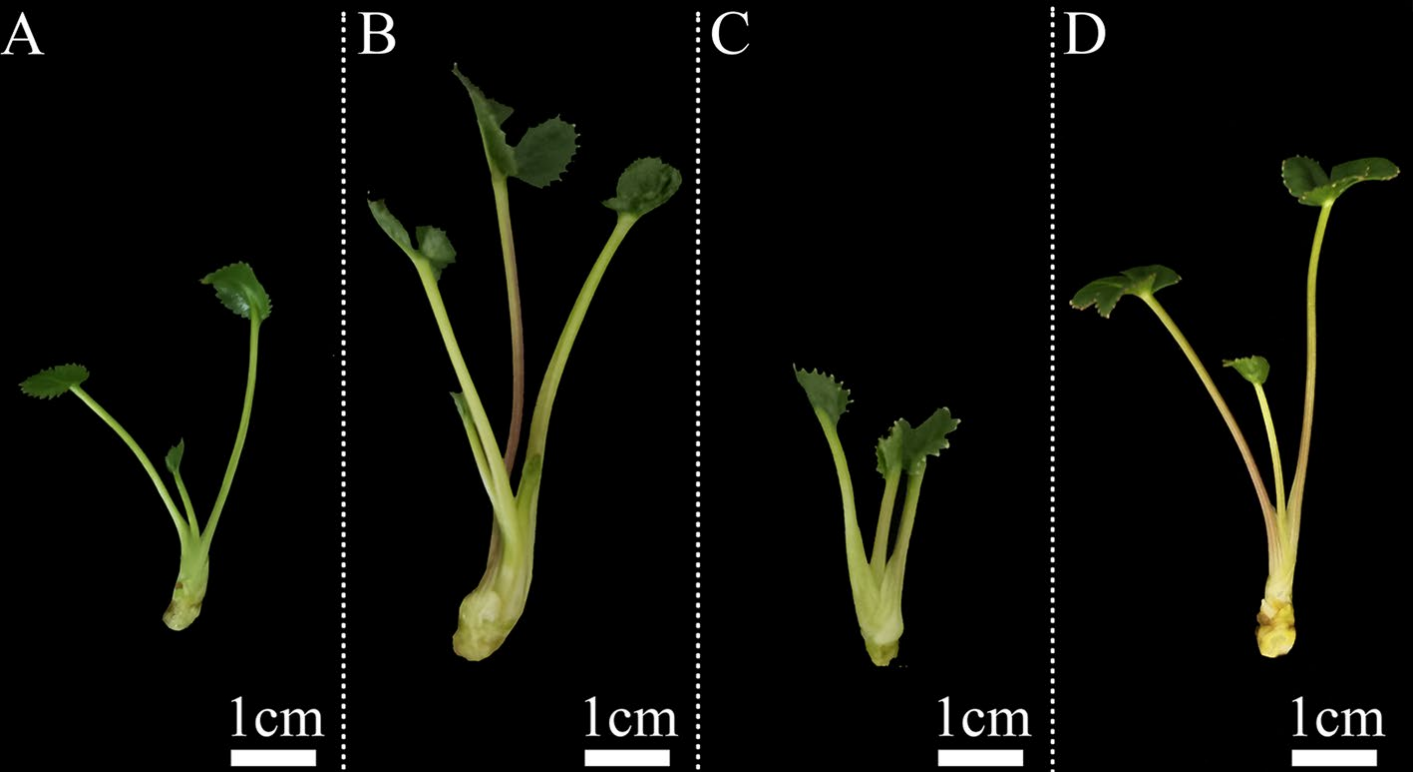
Graded screening of tissue culture plantlets based on morphological characteristics. (**A**) diploid tissue culture plantlets. (**B**) first-grade tissue culture plantlets. (**C**) second-grade tissue culture plantlets. (**D**) third-grade tissue culture plantlets

After three months of culture, a sufficient number of adventitious shoots were formed. Compared to control, five adventitious shoots swelled that may be polyploids were eventually selected. The five adventitious shoots were then regarded as explants for proliferation and were continuously screened by the grading method (Fig. 2). After culture for six months, a total of 42 first-grade plantlets, 69 second-grade plantlets and 84 third-grade plantlets were screened.

### Identification and verification of tetraploid *G. littoralis*

**Fig. 3:**
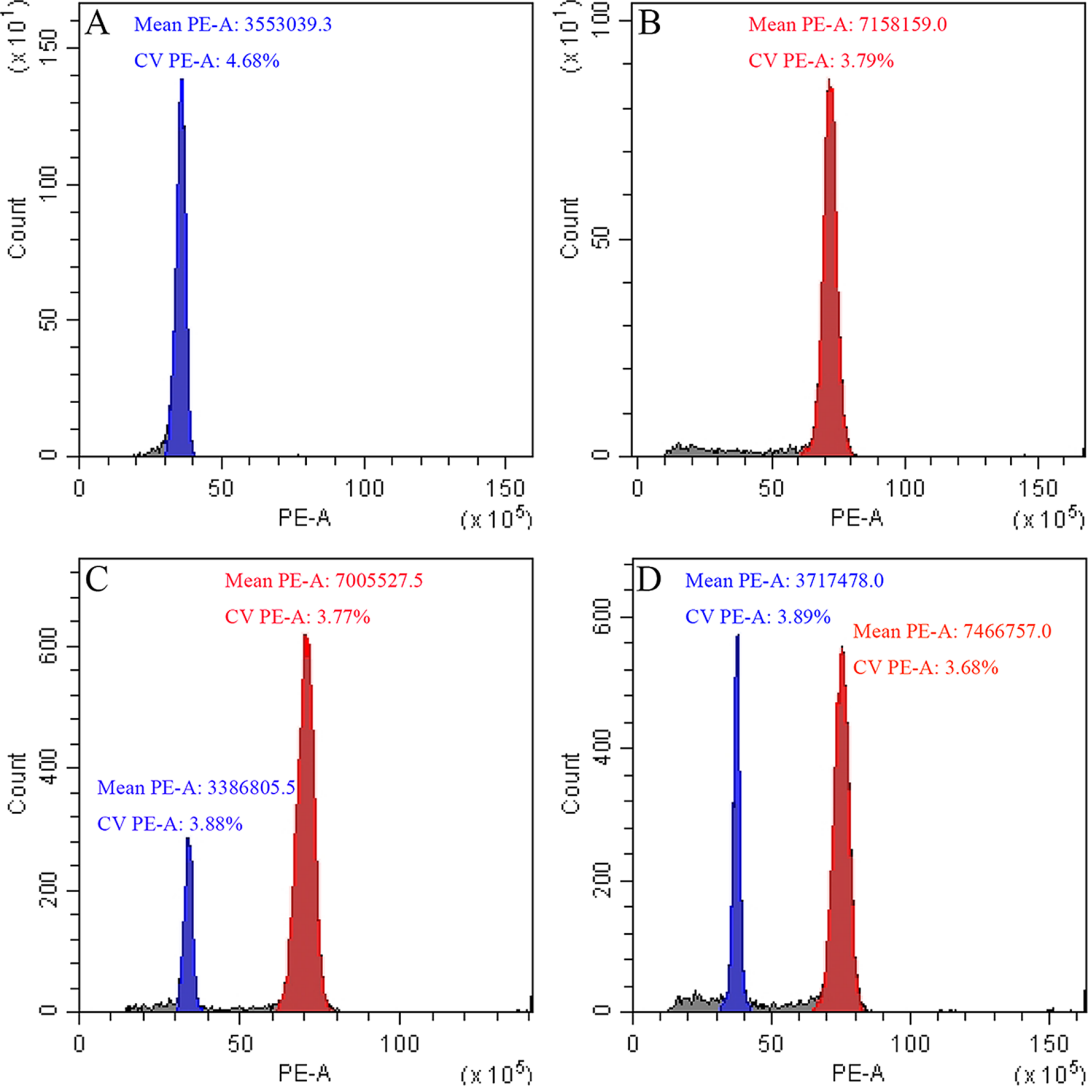
Determination of the ploidy of *G. littoralis* by flow cytometry. (**A**) diploid, (**B**) tetraploid, (**C**) chimera, and (**D**) chimera

Diploid control and 195 tissue culture plantlets screened were checked by flow cytometry assay. Diploid control plants that showed a single peak in the fluorescence channel around 3500,000 (Fig. 3A), and polyploid plants that showed a single peak in the fluorescence channel around 7000,000 were selected as tetraploid because the polyploid had about twice the fluorescence intensity peak of the diploid (Fig. 3B). However, polyploid plants that showed a single peak in each of the fluorescence channels 3500,000 and 7000,000 were selected as chimera. In other words, the presence of two peaks in the assay showed that the regenerated plant was a chimera (Fig. 3C and D). By flow cytometry, it was found that 78.57% of the first-grade plantlets were chimeras and the remaining were diploids. It was observed that 78.26% of the plants in the second-grade plantlets were tetraploids and the remaining were chimeras. No polyploids were detected in third-grade plantlets and all were diploids (Table 1). Overall, we obtained 54 tetraploid plants, all of which were second-grade plantlets and originated from the T1 treatment.

**Table 1:** Number of plantlets by flow cytometry

	No. of tests	No. of tetraploids	No. of diploids	No. of chimeras
First-grade tissue culture plantlets	42	0	9	33
Second-grade tissue culture plantlets	69	54	0	15
Third-grade tissue culture plantlets	84	0	0	0

**Fig. 4:**
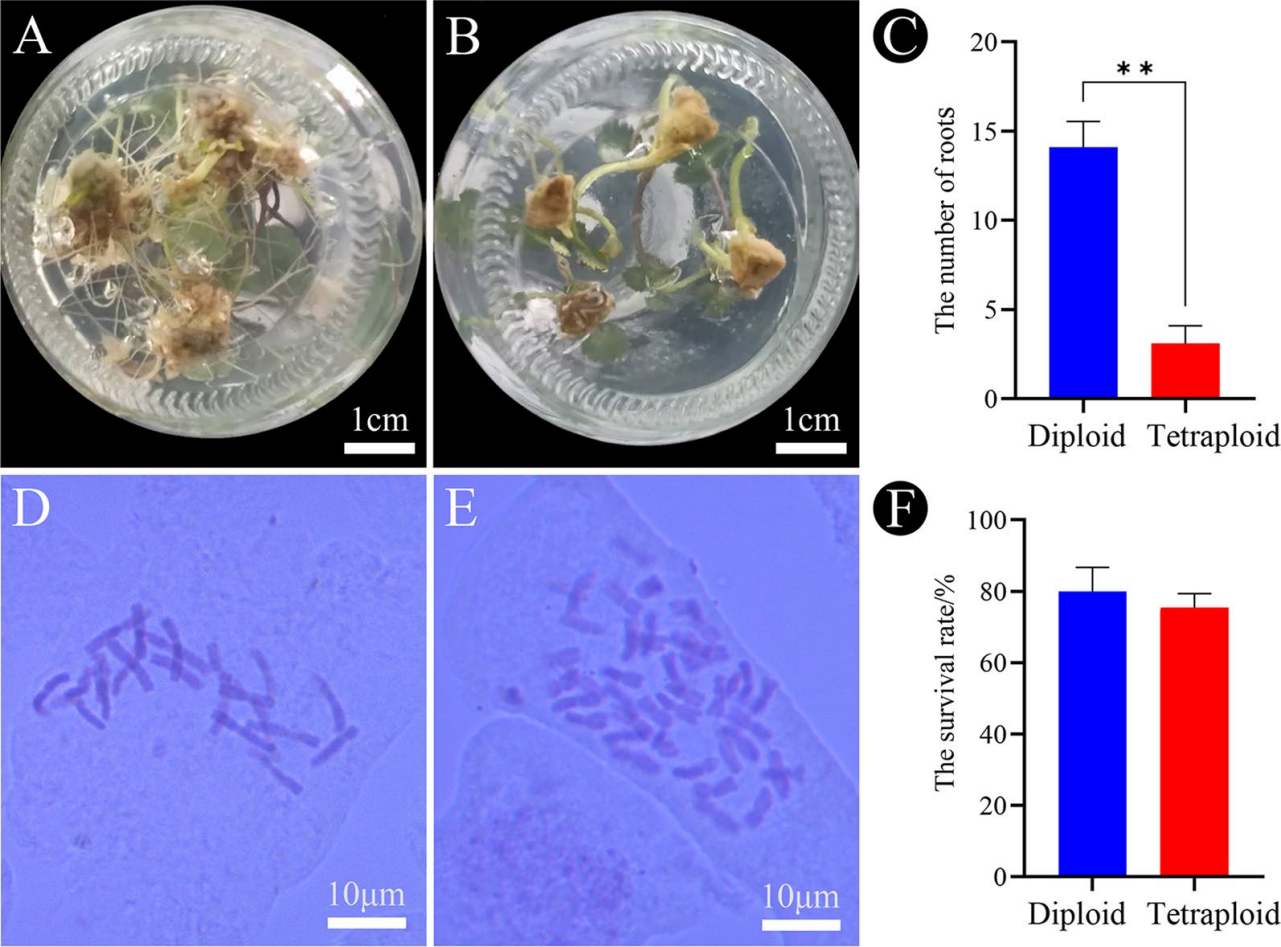
Shoots rooted after one month of culture on the rooting medium. (**A**) diploid, (**B**) tetraploid. (**C**) The number of roots after one month rooting culture. Verification of the ploidy of *G. littoralis* by root tip squash method. (**D**) diploid, (**E**) tetraploid. (**F**) The number of survival rate of plantlets transferred to the substrate after one month

The control and tetraploid plantlets selected by flow cytometry were transferred to the rooting medium. After one month of rooting culture, a large number of roots had already produced in diploids (Fig. 4A) and only a very small number of roots in tetraploids (Fig. 4B), which showed that the rooting rate of tetraploid was slower than that of diploid on the rooting medium (Fig. 4C). The putative tetraploids were ultimately confirmed by somatic chromosome counting. The somatic chromosome number of diploids was 2n = 2x = 22 (Fig. 4D), whereas that of the tetraploids was 2n = 4x = 44 (Fig. 4E). All 54 plantlets were reconfirmed as tetraploids. The diploids and tetraploids were removed from the rooting medium and transplanted into substrate. The survival rate of the diploids transferred to the substrate after one month was 80%, and the survival rate of the tetraploids was 75.5% (Fig. 4F).

### Comparison of morphological characteristics of diploid and tetraploid leaves

**Fig. 5:**
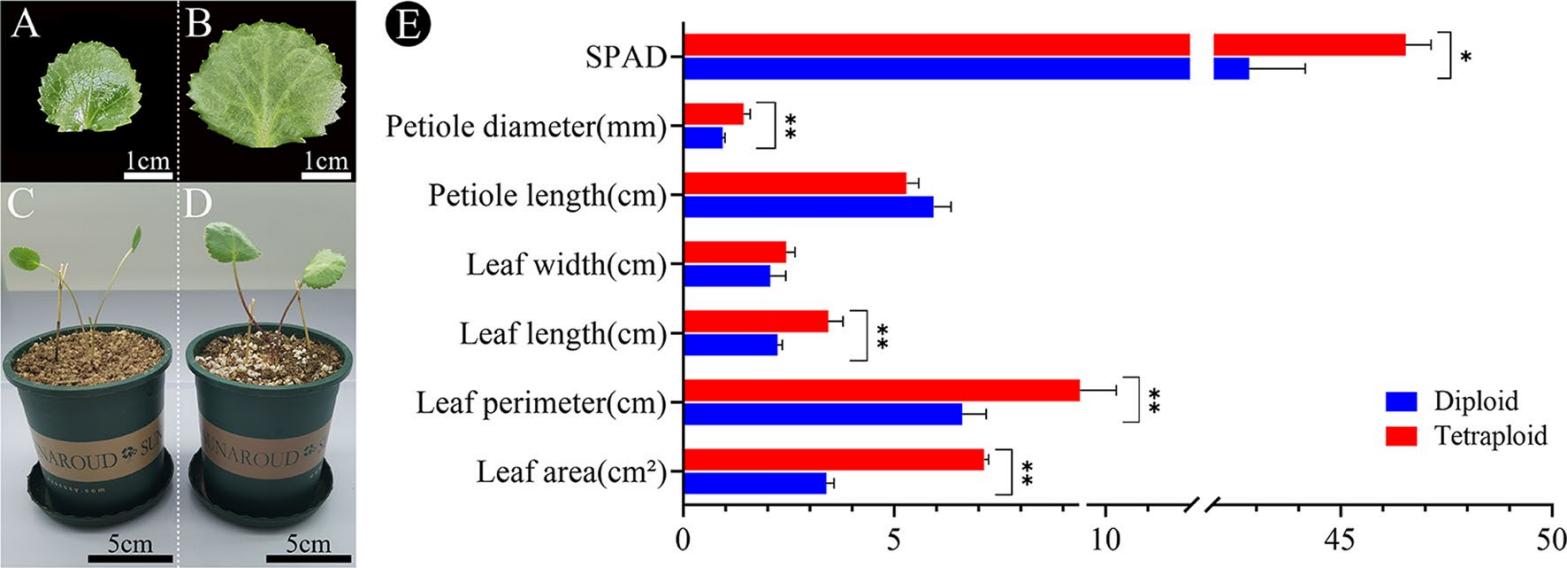
Comparison of leaf morphological traits between diploid and tetraploid. (**A**) diploid, (**B**) tetraploid. Growth of plantlets after a two-month transplantation. (**C**) diploid, (**D**) tetraploid. (**E**) comparison of the morphological characteristics of the leaves of diploid and tetraploid. *p < 0.05, **p < 0.01

The tetraploid *G. littoralis* showed significant phenotypic differences compared to the diploid (Fig. 5). Tetraploid *G. littoralis* had larger leaves, darker green, shorter and thicker petioles, and a downward curving leaf margin (Fig. 5A, C and B, D). Specifically, leaf area, leaf perimeter, leaf length, leaf width, petiole diameter and SPAD of tetraploid *G. littoralis* increased by 110.65%, 42.35%, 53.73%, 18.63%, 53.57% and 8.64%, respectively, while petiole length decreased by 10.84% (Fig. 5E). Moreover, the leaf shape index (leaf length/leaf width) of the tetraploid was 1.41 compared to that of the diploid with a leaf shape index of 1.09.

### Comparison of stomatal characteristics of diploid and tetraploid leaves

**Fig. 6:**
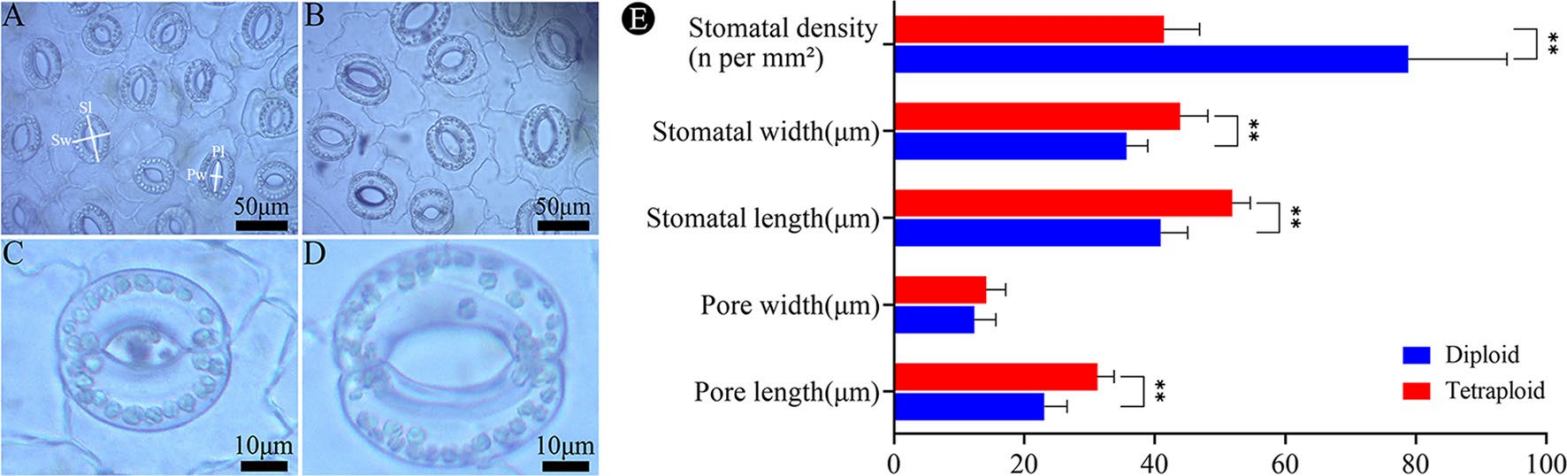
Stomata on leaf abaxial side recorded at 400x magnification. (**A**) diploid, (**B**) tetraploid. Guard cells in the lower epidermis of leaves and chloroplasts in guard cells. (**C**) diploid, (**D**) tetraploid. (**E**) comparison of the stomatal characteristics of the lower epidermis of diploid and tetraploid leaves. Sw: Stomatal width, Sl: Stomatal length, Pw: Pore width, Pl: Pore length. *p < 0.05, **p < 0.01

Compared to the diploid control, the stomatal density of the lower epidermis of tetraploid *G. littoralis* leaves was significantly lower. However, the stomata in tetraploids had larger sizes than the diploids. The length and width of stomata in leaves of tetraploid were significantly larger than those in the diploids (Fig. 6A and B). Furthermore, the number of chloroplasts in the stomata of tetraploid leaves appeared to be higher than those in diploid ones (Fig. 6C and D). Specifically, the stomatal density of tetraploids decreased by 47.5%, stomatal length and width increased by 26.74% and 23.13%, and pore length and width increased by 35.30% and 14.71%, respectively (Fig. 6E).

### Comparison of anatomical characteristics of diploid and tetraploid leaves

**Fig. 7:**
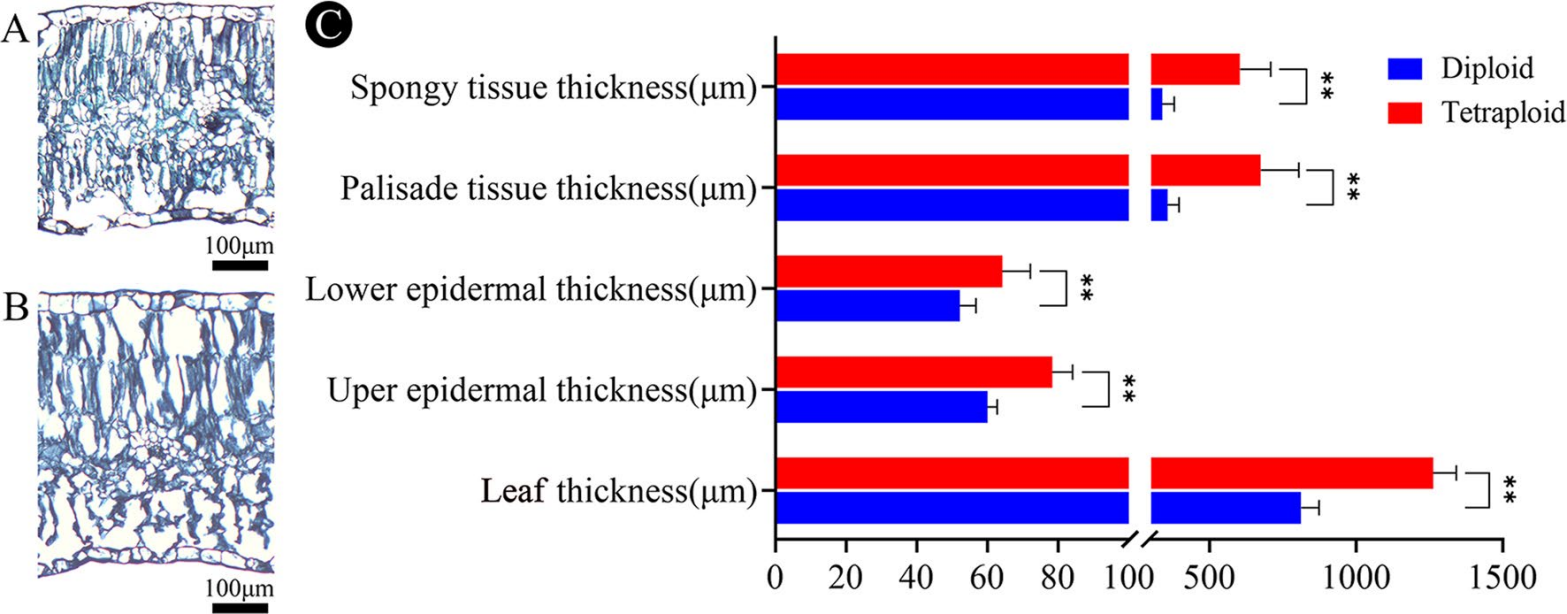
Leaf anatomical characteristics of diploid and tetraploid *G. littoralis*. (**A**) diploid, (**B**) tetraploid. (**C**) comparison of anatomical characteristics of diploid and tetraploid leaves. *p < 0.05, **p < 0.01

The tetraploids of *G. littoralis* demonstrated significant differences in anatomical structures when compared to the diploids (Fig. 7). The thickness of leaves, epidermal tissue, and mesophyll tissue of tetraploids were higher than that of diploids (Fig. 7A and B). Specifically, the thickness of tetraploid leaves, upper epidermis, lower epidermis, palisade tissue, and spongy tissue increased by 55.41%, 30.64%, 22.95%, 89.09% and 78.93%, respectively (Fig. 7C).

### Transcriptome data acquisition

In this study, six libraries were constructed for further RNA-Seq analysis. A total of 31.54 Gb Clean Data was obtained, and the Clean Data of each sample exceeded 5.85 Gb, and the Q30 reached more than 94.67%, as shown in Table 2, which proved that the accuracy of this sequencing data was good and could be used for subsequent analysis. We obtained 57,852 unigenes with a total length of 71,321,171 bp, the longest unigene was 17,083 bp, and the average length was 1232.82 bp. The length of N50 was 2001 bp (Table 3). The correlation heat map analysis detected high correlations between the biological replicates. The results demonstrated that the quality of the transcriptome sequencing was reliable and sufficient for further analysis.

**Table 2:** Statistics of RNA-seq for the diploid and tetraploid samples of *G. littoralis*

Sample	Reads no.	Bases (bp)	Q20(%)	Q30(%)	Clean reads no.	Clean data (bp)	Clean reads (%)	Clean data (%)
GL2_1	40,118,534	6,057,898,634	98.34	94.91	39,690,408	5,988,854,116	98.93	98.86
GL2_2	40,461,444	6,109,678,044	98.24	94.67	40,004,406	6,035,574,132	98.87	98.79
GL2_3	42,817,792	6,465,486,592	98.29	94.77	42,366,240	6,392,861,774	98.95	98.88
GL4_1	39,248,896	5,926,583,296	98.35	94.96	38,849,296	5,857,936,755	98.98	98.84
GL4_2	41,765,016	6,306,517,416	98.25	94.72	41,290,866	6,229,794,283	98.86	98.78
GL4_3	43,004,568	6,493,689,768	98.39	95.05	42,566,474	6,422,122,278	98.98	98.90

*Note* Q20(%): the rate of bases which quality is greater than 20. Q30(%): the rate of bases which quality is greater than 30

**Table 3:** Assembly sequence statistics

	Transcript	Unigene
Total length (bp)	204,404,328	71,321,171
Sequence number	137,104	57,852
Max. length (bp)	17,083	17,083
Mean length (bp)	1490.87	1232.82
N50 (bp)	2118	2001
N50 sequence no.	32,061	11,233
N90 (bp)	710	500
N90 sequence no.	93,653	39,436
GC%	38.59	38.15

### Gene annotation and differential gene analysis

**Fig. 8:**
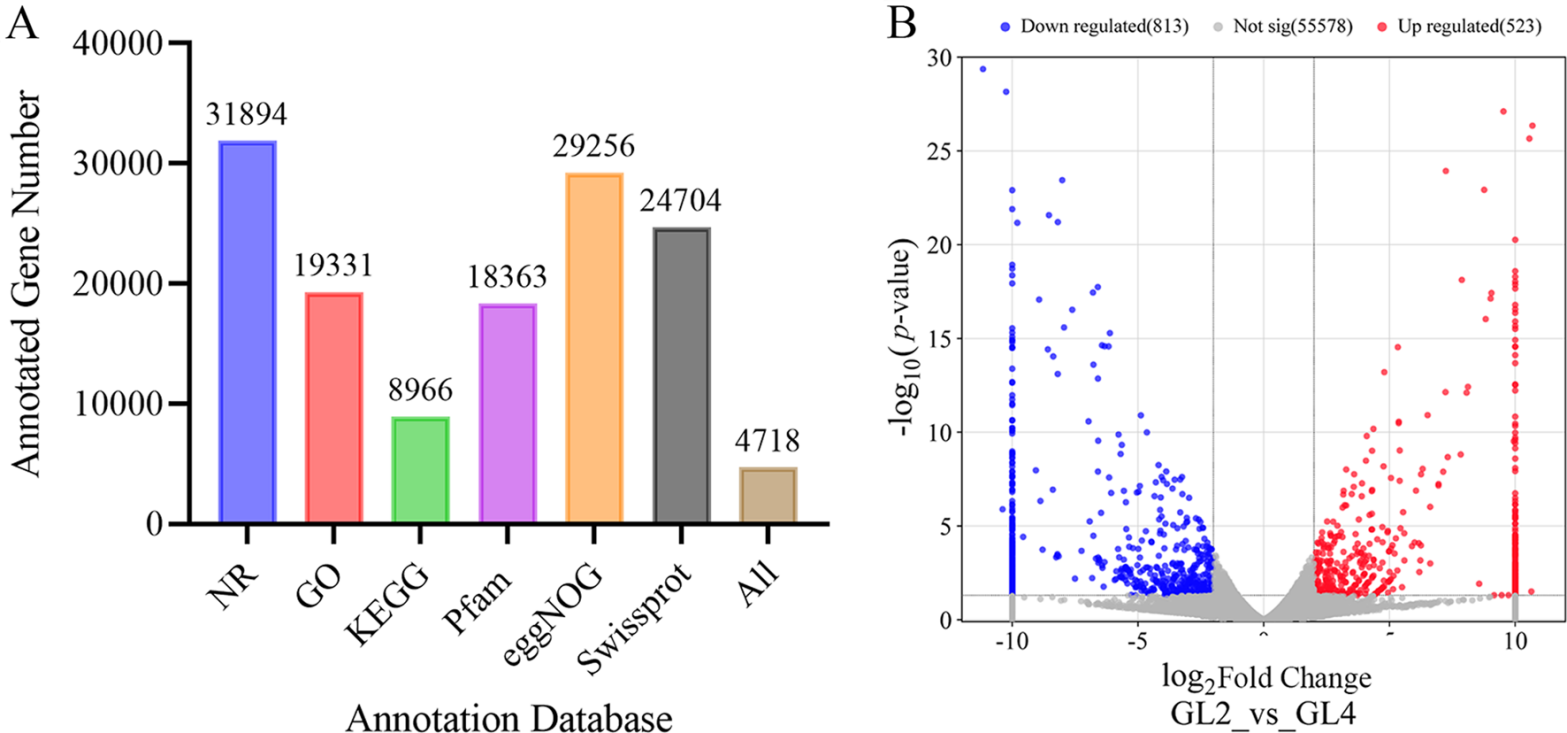
The number of gene annotation and differential expressed gene. (**A**) unigenes database statistics, (**B**) volcano map of differentially expressed genes in diploid (GL2) and tetraploid (GL4)

Gene function annotation of unigenes was performed in six databases (NR, GO, KEGG, Pfam, eggNOG and SwissProt). Among them, the NR database had the largest number of annotated genes: 31,894. As regards to species distribution, these genes matched the known sequence from 457 species and indicated the highest similarity to *Daucus carota* subsp. *sativus*. Specifically, 55.13%, 33.41%, 15.50%, 31.74%, 50.57% and 42.7% of unigenes were successfully annotated in NR, GO, KEGG, Pfam, eggNOG and SwissProt databases, respectively (Fig. 8A). Only 4718 unigenes were annotated in all six databases, representing 8.16% of total unigenes. Moreover, our results showed that only a small number of genes were differentially expressed. A total of 1336 differentially expressed genes (DEGs) were identified between the diploids and the tetraploids, including 523 up-regulated and 813 down-regulated genes (Fig. 8B).

### GO and KEGG analysis of differentially expressed genes (DEGs)

**Fig. 9:**
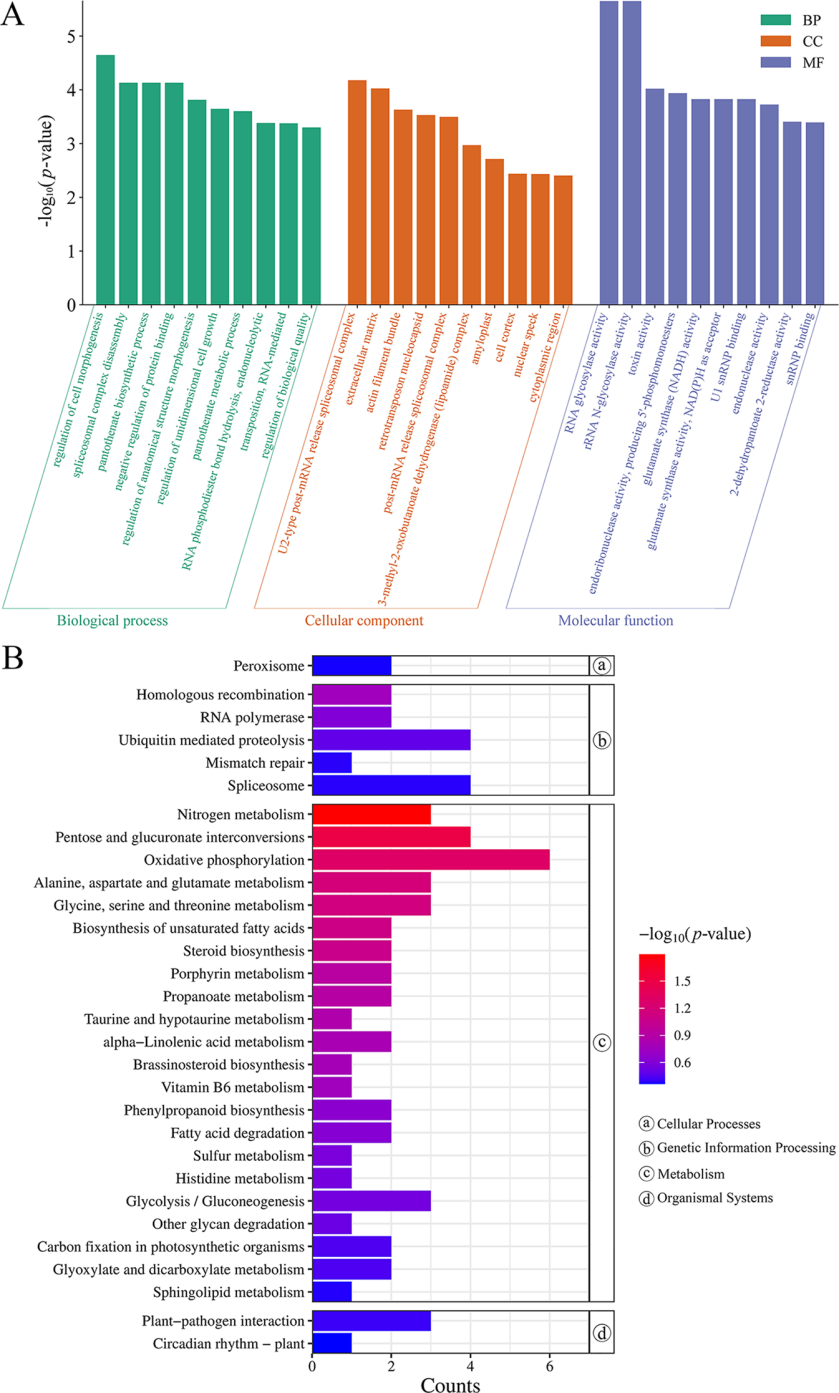
GO and KEGG pathway enrichment analyses of DEGs between diploids and tetraploids. (**A**) GO enrichment analysis with the top 10 of each category. (**B**) KEGG enrichment analysis with the 30 most enriched KEGG pathways. Copyright permission has been granted for related KEGG images

The GO enrichment analyses were used to classify the functions of the annotated DEGs. The DEGs were annotated with GO terms and assigned to biological process (BP), cellular component (CC), and molecular function (MF) (Table S1). The top 10 GO terms (ranked by *p*-value) of each category were listed (Fig. 9A). Compared to the diploids, many DEGs were assigned to phenotype-related GO terms, which included: regulation of cell morphogenesis (GO:0022604), regulation of anatomical structure morphogenesis (GO:0022603), regulation of unidimensional cell growth (GO:0051510), and regulation of biological quality (GO:0065008) under biological process.

**Fig. 10:**
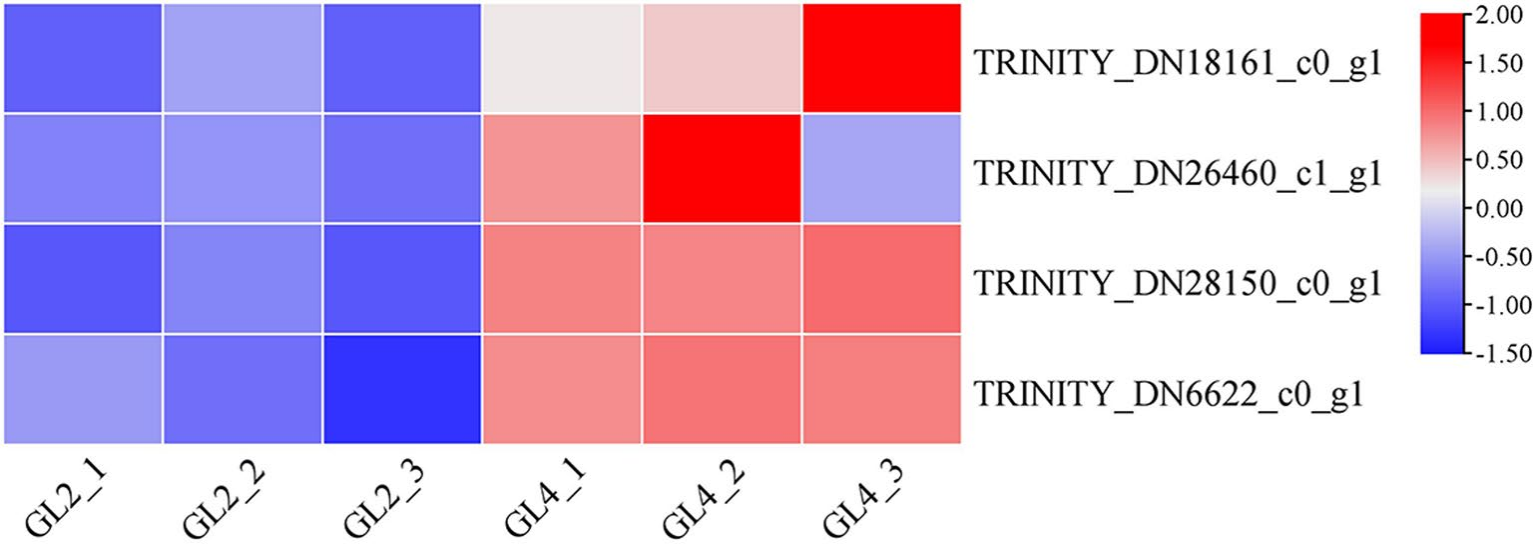
Expression analysis of the DEGs related to growth and development between diploid (GL2) and tetraploid (GL4) *G. littoralis* leaves

KEGG pathway analysis was conducted to categorize gene functions with an emphasis on biochemical pathways that were active in diploid and tetraploid *G. littoralis* leaves. A total of 102 differentially expressed genes were annotated to 58 pathways and assigned to 5 categories, including metabolism, genetic information processing, environmental information processing, cellular processes, and organismal systems (Table S2). The pathways with the largest number of DEGs were metabolic pathways. The number of DEGs related to metabolic pathways was 61, accounting for 59.80%. The KEGG pathways of diploids and tetraploids were enriched and screened to obtain the 30 most enriched signal pathways, including nitrogen metabolism, pentose and glucuronate interconversions, and oxidative phosphorylation (Fig. 9B). We found that several genes related to growth and development were relatively highly expressed in leaves of tetraploid *G. littoralis* such as *TRINITY_DN18161_c0_g1*(casein kinase 1, *CKI*), *TRINITY_DN26460_c1_g1*(histidinol dehydrogenase, *hisD*), *TRINITY_DN28150_c0_g1*(magnesium-dependent phosphatase 1, *MDP1*) *TRINITY_DN6622_c0_g1*(pyruvate, orthophosphate dikinase, *ppdK*) (Fig. 10).

### Quantitative real-time PCR (qRT-PCR) analysis

**Fig. 11:**
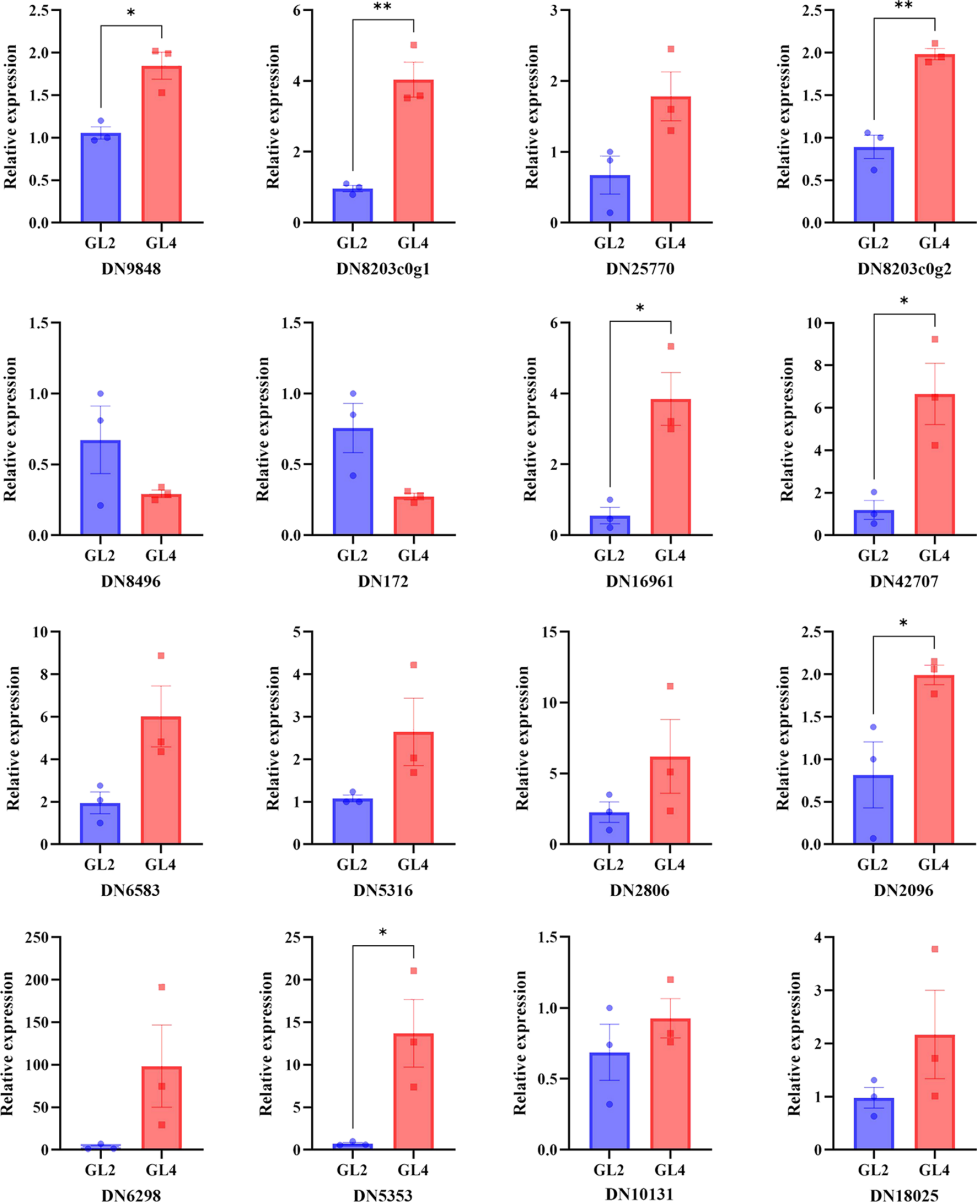
qRT-PCR of 16 DEGs in diploid and tetraploid *G. littoralis* leaves. *p < 0.05, **p < 0.01

To validate the reliability of the RNA-seq results, 16 DEGs were randomly selected, including four up-regulated genes related to morphogenesis, two down-regulated genes related to growth, and ten up-regulated genes related to defense response for qRT-PCR analysis. The expression patterns revealed by qRT-PCR analysis were similar to those obtained by RNA-Seq for the same genes (Fig. 11). These findings indicated that the RNA-seq results of the present study were reliable.

## Discussion

### Induction and screening of tetraploid *G. littoralis*

Polyploidization can produce significant alterations in the genetic traits and phenotypes of plants [18]. Moreover, polyploidy induction in vitro can produce many plantlets in the short time in a limited space [19]. More importantly, it is possible to improve mutagenesis efficiency and reduce chimerism [20]. Therefore, polyploidization can drive plant evolution and domestication through chromosome doubling [21] and it is an effective tool to induce variability in the existing population [22].

Polyploid induction has been successful in numerous plant species [23–25]. Among the various induction methods, colchicine has better effect on polyploid induction in plants [26]. To the best of our knowledge, this is the first report on successful induction of tetraploid in *G. littoralis* in vitro. In the previous studies, colchicine was used to induce chromosome doubling, but different plants require different concentrations and treatment time. The explants used in our study were also different from those used in previous studies. In the previous studies, shoot tips [22], pollens [25], and leaf tissues [27] were used as explants for the induction of polyploidy in vitro. In this study, seeds as explants were effective for polyploidy induction of *G. littoralis* in vitro. The achievement of desired outcomes necessitates the implementation of appropriate conditions, whether through in vitro conditions or direct application on the plants. Direct induction of polyploidy in vivo is relatively simple, but difficult to isolate chimeras because it is very difficult to double the chromosomes of all cells.

Due to the randomness of mutations caused by asexual polyploidy, plant tissues may contain hybrids or chimeras. In this study, we also found chimeras as reported in other different crops [22]. Generally, colchicine disrupts mitosis by preventing microtubule polymerization, resulting in inhibition in polar migration of chromosomes at anaphase. The treatment of explants with colchicine may cause some chromosomal aberration, resulting in the formation of aneuploid population. Due to the increased ploidy, meiosis of asexual polyploids is unstable, and the number of polyploid cells in the chimera may decrease during plant growth, causing them to degenerate or even return to diploids in the process of reproduction [26, 27], which defeats the purpose of breeding. Therefore, selecting stable tetraploid is extraordinarily important for breeding polyploid varieties. To achieve a higher level of ploidy purity, it is necessary to engage in several rounds of thorough screening and validation.

Interestingly, in our study, no tetraploid except mixoploids was detected in first-grade plantlets with thick and long petioles. However, most of the second-grade plantlets with the shortest and slightly thinner petioles were tetraploids. The third-grade plantlets with slender petioles did not double their chromosomes. We speculated that some cells in the first-grade seedlings might have undergone multiple doubling of chromosomes. The presence of polyploid cells in chimeric plants may lead to onset of prominent phenotypic change. The third-grade plantlets are derived from undoubled cells, so they are diploid, and with thin and long petioles. Petiole thickness of the second-grade plantlets is between the two, but the length is the shortest, which is consistent with the dwarfed homologous tetraploid reported in black locusts by Wu et al. And they found several DEGs and endogenous hormones related to plant growth and development by metabolite analysis and comparative transcriptomics, and showed plant hormone signaling and the circadian rhythm pathway result in dwarfism [18].

### Phenotypic and transcriptomic analysis of tetraploid *G. littoralis*

The tetraploid commonly displays some superiority in morphological characteristics such as leaf area in Wallflower (*Erysimum cheiri*) [28], leaf thickness in Grape (*Vitis vinifera*) [29], and the length and width of stomata in *Hippeastrum papilio* [30]. These traits facilitate the sequestration of carbon through photosynthesis [31]. In this study, plant growth and development as well as histological differences of leaves between the diploid and its autotetraploid were investigated. The tetraploids obtained showed several morphological differences compared with the diploid ones on the functional traits of leaves after a two-month transplantation period. In line with previous studies, larger leaves, thicker mesophyll tissue, higher chlorophyll content, thicker petioles of tetraploids might have facilitated better photosynthesis and enhanced the mechanical resistance to environmental changes. Compared to diploids, the superior agronomic traits of tetraploids could have enhanced their chance of thriving in new environments and furthermore improve the yield and quality of agricultural production [32]. It will be of great value in the selection and breeding of new varieties in the future. In this study, compared to the diploid control, the stomatal apparatus of tetraploid *G. littoralis* leaves had significantly lower density, larger sizes, and contained guard cells with more chloroplasts. Whether these traits are more beneficial to tetraploid is worth further investigating.

An autopolyploid is different from its diploid progenitor, as the DNA content and dosage effect of each gene are doubled [33]. Both effects increase the size of cells and organelles by altering gene expression, leading to phenotypic variation [34, 35]. Transcriptome sequencing data analysis showed that differentially expressed genes (DEGs) were significantly enriched in biological process of biomineral tissue development, biomineralization, regulation of cell morphogenesis, and regulation of anatomical structure morphogenesis, which are closely related to plant cell structure and growth. And several genes related to them were highly expressed in leaves of tetraploid *G. littoralis* such as *CKI*, *PPDK*. *CKI* gene in tetraploid *G. littoralis* was significantly up-regulated. Previous study in cotton had found that *CKI* gene was underwent amplification with cotton tetraploid event and it was involved in the response to light signals [36]. In *Arabidopsis*, the Casein kinase 1 family regulates PRR5 and TOC1 in the circadian clock, impacting crucial plant processes like photosynthesis, cell elongation, stress responses, and flowering, with potential effects on biomass or yield [37–40]. We speculated that the higher expression of *CKI* genes in tetraploid *G. littoralis* might be related to cell elongation, so as to regulate size and thickness of leaves, stomatal size. But systematic identification and characteristics of *G. littoralis CKI* gene will be needed in the future. *PPDK* gene encodes both a cytosolic and chloroplastic protein and it has been shown that *PPDK* gene is required for plant establishment [41], and positively correlated with the increased accumulation of photosynthetic pigment [42]. The foxtail millet transferred to the C4 gene *PPDK* enhanced photosynthesis and yield to a greater extent [43]. We assumed that the tetraploid *G. littoralis* might have a high yield.

## Conclusions

In this study, we induced and screened tetraploids of *G. littoralis* by chromosome doubling technique, and compared the morphological and cytological differences between diploid and autotetraploid plants. The transcriptome sequencing was performed to analyze the differentially expressed genes that are associated with the phenotypic changes associated with the tetraploid plants. The obtained tetraploids of *G. littoralis* exhibited superior agronomic traits, especially leaf traits. By transcriptome sequencing, several up-regulated DEGs were found related to growth and development in tetraploid *G. littoralis* such as *CKI*, *PPDK*, *hisD* and *MDP1*. These results suggest that tetraploids of *G. littoralis* may serve as a potential genetic resource to provide valuable agronomic traits to facilitate molecular breeding of *G. littoralis* varieties.

### Methods

#### Materials

Mature, full and uniform *G. littoralis* fruits were collected from the Germplasm Resource Nursery of Yantai University (37°28′24.02"N, 121°27′51.85"E), and identified by Professor Fuhua Bian from the College of Life Science, Yantai University. The fruits were placed in wet sand at 4 °C for three months for preservation. Then, the fruits were washed and the pericarp was removed for acquiring clean seeds. The seeds were placed in petri dishes in a culture room at 25 ± 2℃ to germinate in the dark, and considered as germinated when radicle exceeded 1 mm.

#### Polyploidy induction of *G. littoralis*

Firstly, the germinated seeds were sterilized, the sterilization process included: soaking in 75% alcohol for 30 s, washing 3 times with sterile water, soaking in 0.1% mercuric chloride for 12 min, washing 5 times with sterile water, during which they were shaken constantly to make them uniformly sterilized. The sterilized seeds were soaked in sterile colchicine solution for different treatment concentrations and durations. Colchicine (purity ≥ 98%) was purchased from Macklin (Shanghai, China). The powder of colchicine was dissolved in a minimum volume of ethanol (90%), brought to the desired concentration (w/v) by adding filter sterilized water. Based on the results of the pre-experiments, four treatments were performed including T1, T2, T3 and CK (control). T1, T2, and T3 were with the concentrations of 0.05% (w/v) and 48 h, 0.1% (w/v) and 24 h, 0.15% (w/v) and 16 h, respectively. The seeds that had not been treated with the colchicine solution were regarded as controls. Then the seeds were washed with sterile water for 3 times and inoculated into the proliferation medium (MS + 2.0 mg/L 6-BA + 0.3 mg/L NAA). All operations were conducted on clean workbench. Lastly, they were placed in the culture room at 25 ± 2℃, 12 h darkness and 12 h light, with an intensity of 2000 lx from LED grow lights. The survival rates were assessed by counting the number of surviving seeds. The growth of adventitious shoots was observed and the focus was on shoots that showed swelling. The swelling shoots were considered to be the imminent polyploid *G. littoralis*.

#### Preliminary grading and screening of *G. littoralis*

For the first three months, a sufficient number of adventitious shoots were cultured. Polyploidy that showed swelling shoots compared with diploid shoots was initially selected. Next, based on plant height and petiole diameter, the tissue culture plantlets were graded and screened. The plantlets with 7–8 cm in height and petiole diameter of 2–2.5 mm were regarded as Grade 1, 3–4 cm in height with petiole diameter of 1.5–2 mm as Grade 2, 6–7 cm in height with petiole diameter of 1–1.5 mm as Grade 3. The rest plantlets were discarded. To obtain pure ploidy plants, the tissue culture plantlets were screened around three months during the incubation process.

#### Determination of the ploidy of *G. littoralis* by flow cytometry

Ploidy analysis was performed on screened tissue culture plantlets by flow cytometry. Nuclear suspension solution was prepared as described previously [44], except that we used an external standard method. Approximately a 20 mg sample of young leaves was placed into clean, pre-cooled culture dishes containing 2 mL of OTTO Ι nuclei extraction buffer (Yuanye, Shanghai, China) and chopped with a sharp blade. The mixture was then filtered through a 30 μm filter membrane into a 1.5 ml centrifuge tube after 5 min on ice. The filtrate was centrifuged at 1,000 g for 5 min at 4 °C, and the supernatant was discarded. Then, 100 µL of OTTO Ι buffer, 400 µL of OTTO II buffer (Yuanye, Shanghai, China), and 500 µL of propidium iodide staining solution (Servicebio, Wuhan, China) were added sequentially. The sample was stained for 20 min at 4 °C, protected from light, and then filtered through 40 μm nylon meshes. Nuclear suspension was analyzed with a Beckman CytoFLEX flow cytometer (Beckman CytoFLEX, USA) and the corresponding CytExpert software. Ten thousand particles were recorded for each sample.

#### Verification of the ploidy of *G. littoralis* by root tip squash method

Diploid controls and tissue culture plantlets that were determined to be tetraploid by flow cytometry were transferred to rooting medium (MS + 0.5 mg/L NAA) for further cultivation. The ploidy of tissue culture plantlets was verified by root tip squash method [45]. When the roots were 1–2 cm in length, the root tips were collected at 10:00 a.m., pretreated with 0.1% colchicine solution for 3 h at 4 °C, washed with distilled water, and then fixed in FAA solution (formalin: glacial acetic acid: 70% ethanol = 5: 5: 90, v: v: v) for 24 h at 4 °C. The fixed root tips were washed with distilled water and dissociated with 1 mol/L hydrochloric acid in a constant temperature water bath at 60℃ for 9 min. The root tip (1 mm) was cut onto a glass slide, and one phenol magenta solution drop was added before covering it with a coverslip. The sample was stained for 40 min after being pressed. The chromosomes were observed using a light microscope (Motic-B5, Shanghai, China) at 1000x magnification and photographed using the Motic Images Plus 3.0 (×64) software.

#### Evaluation of the phenotypic characteristics of diploid and tetraploid *G. littoralis*

After about 40 days of rooting culture in the rooting medium, the diploid and tetraploid plantlets were planted in the substrate (sand: coconut bran: perlite = 1: 2: 3, v: v: v) at 25 ± 2℃ for two months. The petiole length and diameter were measured using a straightedge and a vernier caliper, respectively. The area, perimeter, length, and width of leaves were measured using a leaf meter (Yaxin-1241, Beijing, China) and the relative content of chlorophyll (SPAD) was determined using a chlorophyll meter (Yaxin-1260, Beijing, China). In addition, at 9:30 a.m., the lower epidermis of the leaf blade was torn with tweezers and placed on a glass slide with water droplets, covered with a cover slip [27] and observed under a Motic-B5 light microscope at a magnification of 400x. Three different fields of view were taken for each section to count the number of stomata. The sizes of three stomata per field of view were measured randomly using the Motic Images Plus 3.0 (×64) software. The anatomical structures of leaves were analyzed by paraffin-embedded tissue Sections [46]. Six samples, each from diploids and tetraploids plants, were randomly selected from six independent plants for each experimental analysis.

### RNA sequencing

Fresh and healthy leaves were collected from diploid and tetraploid *G. littoralis* plants grown in the substrate for two months, quickly frozen in liquid nitrogen, and then stored in a refrigerator at -80 °C before RNA extraction. Sampling was performed at noon, with three biological replicates for each sample group. Total RNA was extracted using the TransZol Up Plus RNA Kit (Tiangen, Shanghai, China), and then the concentration, quality and integrity were determined using a NanoDrop 2000 spectrophotometer (Thermo Scientific, MA, USA). Sequencing libraries were constructed using the TruSeq RNA Sample Preparation Kit (Illumina, San Diego, CA, USA). Sequencing was performed on a NovaSeq 6000 platform (Illumina) by Shanghai Personal Biotechnology Cp. Ltd.

### *De novo* assembly and gene functional annotation

We used fastp (0.22.0) software filter the sequencing data to get high quality sequence (clean reads) for further analysis. Q20 and Q30 are quality scores that measure the percentage of bases with sequencing error rates of less than 1% and 0.1%, respectively. Higher Q20 and Q30 scores indicate better sequencing quality and accuracy. Trinity (v2.15.1) software was employed for *de novo* assembly of clean reads into transcripts for the transcriptome sequencing project without reference genome. The assembled transcripts were saved in FASTA format, with the longest transcript for each gene extracted as the representative sequence (unigene). The database used in gene function annotation includes NR (NCBI non-redundant protein sequences), GO (Gene Ontology), KEGG (Kyoto Encyclopedia of Genes and Genome), eggNOG (evolutionary genealogy of genes: Non-supervised Orthologous Groups), Swiss-Prot, Pfam.

### Differential expression genes (DEGs) analysis

RSEM (v2.15) statistics was used to compare the Read Count values on each gene as the original expression of the gene and used FPKM to standardize the expression. DESeq2 R package (1.38.3) was used to analyze difference expression of genes with conditions as follows: expression difference multiple |log_2_FoldChange| > 2, significant *p*-value < 0.05. R language Pheatmap (v1.0.12) software package was used to perform bi-directional clustering analysis of all different genes.

We mapped all the genes to terms in the Gene Ontology database and calculated the numbers of differentially enriched genes in each term. GO term enrichment analysis of DEGs was carried out using the topGO R package (v2.50.0). ClusterProfiler (v4.6.0) software was used to carry out the enrichment analysis of the KEGG pathway of differential genes, focusing on the significant enrichment pathway with *p*-value < 0.05. The KEGG pathway enrichment was used to identify major biochemical and signal transduction pathways where the DEGs participated [47–49].

### Quantitative real-time PCR analysis of DEGs

To check the accuracy of transcriptomic analysis, some up- or down-regulated DEGs involved in leaf development were selected for quantitative real-time PCR (qRT-PCR) assays. The primers were designed using Primer 5.0 software, which were listed in Table S3. The *G. littoralis* actin gene was used as a reference gene [50]. Total RNA was extracted from diploid and tetraploid *G. littoralis* leaves using the Trizol reagent (Invitrogen, Carlsbad, CA, USA). The qualified and quantified total RNA was reversely transcribed into cDNA using the PrimeScript TM 1st STAND cDNA Synthesis Kit. PCR reactions were performed on a MA-6000 real-time thermal cycler, and the reaction procedures were as follows: 95 °C for 5 min, followed by 40 cycles of 95 °C for 15 s, and 60 °C for 30 s. The relative expression levels of the genes were calculated using the 2^−∆∆Ct^ method [51]. Three biological replicates were performed for each gene.

### Statistical analysis

All data were expressed as mean ± standard deviation. Microsoft Excel and GraphPad Prism 9.1 software were used for data analysis. The data was subjected to an analysis of variance. Student’s t test was used to compare differences between the control and treated groups. A significant difference relative to the control was determined at *p < 0.05 or **p < 0.01.

### Electronic supplementary material

Below is the link to the electronic supplementary material.

Supplementary Material 1

Supplementary Material 2

Supplementary Material 3

## Data Availability

The RNA-seq dataset was deposited in the NCBI Sequence Read Archive (SRA, www.ncbi.nlm.nih.gov/Traces/sra) with accession number PRJNA1049611 (www.ncbi.nlm.nih.gov/sra/PRJNA1049611). All other datasets generated in this study are available within the paper and its additional files.
